# Supplementary material for: Pre-Amplification of Cell-Free DNA: Balancing Amplification Errors with Enhanced Sensitivity
Source: Biomolecules. 2025 Jun 17;15(6):883. doi: 10.3390/biom15060883 (PMC12191314; doi:10.3390/biom15060883)
Supplement: Supplementary file 1 [file biomolecules-15-00883-s001.zip › biomolecules-3653348-supplementary.pdf]

Supplementary Tables

Supplementary Table S1. Summary of Patient Cohort

**Characteristics**	**No. of Patients (N=39)**
Age (years), mean (range)	66 (32-94)
Sex, n (%)	
Male	24 (62)
Female	15 (38)
Stage (AJCC 8th edition), n (%)	
Stage III	10 (26)
Stage IV	29 (74)
Tissue mutation profile, n (%)	
BRAF	17 (44)
NRAS	11 (28)
TERT	9 (23)
Others	2 (5)
Total cfDNA yield (ng/ml plasma), n (%)	
Mean (range)	12.0 (4.3-46.8)
Plasma Timepoint[a], n (%)	
Pre-treatment	9 (23)
Early during treatment	30 (77)
Systemic therapy, n (%)	
Combination anti-PD-1 and anti-CTLA-4	24 (63)
Single agent anti-PD-1	15 (38)
Recurrence/disease progression, n (%)	
Yes	18 (46)
No	21 (54)

[a]Plasma samples were collected before treatment (pre-treatment) or 6-12 weeks from treatment commencement (early during treatment).

Supplementary Table S2. List of probes employed in ddPCR analysis

Assay Name	Probe Fluorophore	Supplier	Wet Lab Validated	Assay ID / Sequence
BRAF p.D594N c. 1780G>A	FAM/HEX	Bio-Rad	No	dHsaMDS2515962
BRAF p.K601E c.1801A>G	FAM	Bio-Rad	No	dHsaIS2504434
BRAF WT for p.K601E c.1801A	HEX	Bio-Rad	No	dHsaIS2504435
BRAF p.V600E c.1799T>A	FAM/HEX	Bio-Rad	Yes	dHsaMDV2010027
BRAF p.V600K c.1798_1799GT>AA	FAM/HEX	Bio-Rad	Yes	dHsaMDV2010035
BRAF p.V600R c.1798_1799GT>AG	FAM	Bio-Rad	Yes	dHsaCP2000037
BRAF WT for p.V600R c.1798_1799GT>AG	HEX	Bio-Rad	Yes	dHsaCP2000038
CDKN2A p.P114L c.341C>T	FAM	IDT	No	5'-/56-FAM/CGT CTG +C+C +C GTG GAC/3IABkFQ/-3'
CDKN2A WT for p.P114L c.341C>T	HEX	IDT	No	5'-/5HEX/CGT CTG +C+T+C GTG GAC/3IABkFQ/-3'
GNA11 p.Q209L c.626A>T	FAM/HEX	Bio-Rad	Yes	dHsaMDV2010049
IDH p.R132H c.395G>A	FAM	Bio-Rad	Yes	dHsaCP2000055
IDH WT for p.R132H c.295G>A	HEX	Bio-Rad	Yes	dHsaCP2000056
NRAS p.Q61H c.183A>T	FAM/HEX	Bio-Rad	No	dHsaMDS218656461
NRAS p.Q61K c.181C>A	FAM/HEX	Bio-Rad	Yes	dHsaMDV2010067
NRAS p.Q61L c.182A>T	FAM/HEX	Bio-Rad	Yes	dHsaMDV2010069
NRAS p.Q61R c.182A>G	FAM/HEX	Bio-Rad	Yes	dHsaMDV2010071
NRAS p.Q61R c.181_182CA>AG	FAM/HEX	Bio-Rad	No	dHsaMDS424048678
RAC1 p.P29S c.85C>T	FAM/HEX	Bio-Rad	No	dHsaMDS2513840
TERT c. 1-124C>T	FAM/HEX	Bio-Rad	No	dHsaEXD20945488
TERT c. 1-146C>T	FAM/HEX	Bio-Rad	No	dHsaEXD85215261
TP53 p. K120M c.359A>T	FAM/HEX	Bio-Rad	No	dHsaMDS2516044
TP53 p.R282W c.844C>T	FAM/HEX	Bio-Rad	Yes	dHsaMDV2516902
TP53 p.Y220C c.659A>G	FAM/HEX	Bio-Rad	Yes	dHsaMDV2510536
TP53 p. Y236C c.707A>G	FAM/HEX	Bio-Rad	Yes	dHsaMDV2516916

WT, wild type; + indicates locked nucleic acid in probe sequences

Supplementary Table S3. PCR amplicons tested for TOP-PCR amplification efficiency

Gene Target	Amplicon Size (bp)	%GC content[a]	hg19 coordinates[b]	Assay ID[c]
BRAF	91	40	chr7:140453075-140453197	dHsaCP2000028
CDKN2A	75	76	chr9:21970956-21971078	dHsaMDV2516934
NRAS	64	46	chr1:115256469-115256591	dHsaMDS672933519
TERT	88	84	chr5:1295207-1295294	dHsaEXD85215261
TP53	68	52	chr17:7577513-7577635	dHsaMDV2516916

[a]% GC content based on sequence data defined by genome coordinates

[b]Sequence coordinates around each amplicon provided

[c]Bio-Rad Laboratories Assay ID

Supplementary Table S4. Detection of ctDNA in stage III melanoma patients with and without TOP-PCR amplification

Patient[a]	Mutation screened	Unamplified cfDNA yield (ng)	Amplified cfDNA yield (ng)[b]	Unamplified MT droplets[c]	TOP-PCR MT droplets[c]
**45141**	BRAF K601E	27.4	160.8	0	16
**48810**	NRAS Q61L	26.0	140.4	0	18
**52021**	BRAF V600K	34.2	166.8	3	23
**48102**	BRAF V600E	31.0	126.0	3	37
**52521**	BRAF V600E	37.2	207.6	1	21
**51902**	NRAS Q61R	51.4	148.2	4	25

[a]These six patients were included in the Chan et al. (2024) study; however, the cfDNA yields, amplification parameters, and ddPCR data were not previously reported.

[b]20 ng input cfDNA was amplified for five cycles. cfDNA yield (ng) was quantitated using Qubit High Sensitivity dsDNA kit and Qubit 3.0 Fluorometer.

[c]Number of FAM^+/HEX^- mutant (MT) droplets are shown.

Supplementary Table S5. TOP-PCR mutation errors in ddPCR runs

Assay Name	Assay ID	Wet lab validated	ddPCR runs with errors/ddPCR runs[a]	
			5 cycles[b]	7 cycles[b]
BRAF D594N c. 1780G>A	dHsaMDS2515962	No	ND	0/3
BRAF K601E c.1801A>G	dHsaIS2504434/5	No	0/8	0/1
BRAF V600E c.1799T>A	dHsaMDV2010027	Yes	4/11	13/18
BRAF V600K c.1798_1799GT>AA	dHsaCP2000035/6	Yes	1/3	0/3
BRAF V600R c.1798_1799GT>AG	dHsaCP2000037/8	Yes	ND	0/1
IDH1 R132H c.395G>A	dHsaCP2000055/6	Yes	ND	1/1
NRAS Q61H c.183A>T	dHsaMDS218656461	No	ND	0/2
NRAS Q61H c.183A>C	dHsaMDV2510578	Yes	ND	0/2
NRAS Q61K c.181C>A	dHsaMDV2010067	Yes	0/7	2/9
NRAS Q61L c.182A>T	dHsaMDV2010069	Yes	0/7	1/2
NRAS Q61R c.182A>G	dHsaMDV2010071	Yes	0/6	0/1
NRAS Q61R c.181_182CA>AG	dHsaMDS424048678	No	0/3	ND
RAC1 P29S c.85C>T	dHsaMDS2513840	No	ND	0/2
TERT C228T c.1-124C>T	dHsaEXD20945488	No	ND	1/5
TERT C250T c.1-146C>T	dHsaEXD85215261	No	0/3	0/5
TP53 K120M c.359A>T	dHsaMDS2516044	No	0/3	ND
TP53 R282W c.844C>T	dHsaMDV2516902	Yes	ND	1/1
TP53 Y220C c.659A>G	dHsaMDV2510536	Yes	ND	0/2

[a]The number of negative control reactions (cfDNA from cultured primary neonatal HDF1314 human dermal fibroblasts) that displayed FAM^{+}/HEX^{-} mutant droplets for the indicated mutation.

[b]Number of TOP-PCR amplification cycles; ND, not determined. 20 ng input was amplified for five PCR cycles and 20 ng input was amplified for seven PCR cycles.

Supplementary Figures

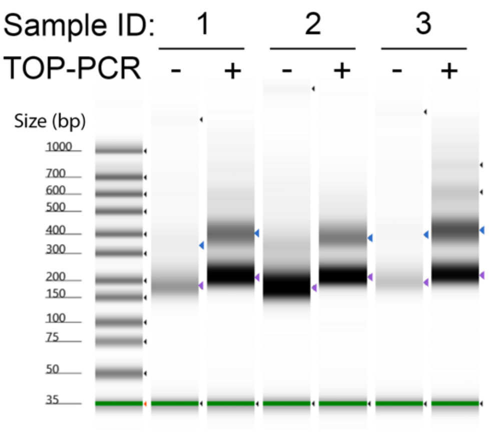

ID	PRE TOP-PCR		POST TOP-PCR	
	DNA (ng/µl)	%cfDNA[a]	DNA (ng/µl)	%cfDNA[a]
1	1.676	92	8.78	96
2	7.480	98	8.63	99
3	0.882	88	6.72	94

[a]% cfDNA is the 100-700 bp DNA fraction

Supplementary Figure S1. TapeStation electrophoresis of melanoma cfDNA pre (-) and post (+) TOP-PCR amplification, shown without intensity scaling

cfDNA derived from three melanoma patients separated using the Agilent TapeStation 4150. Each sample was amplified using 20ng of input cfDNA (five cycles of amplification). The samples are shown without intensity scaling to preserve true differences in band signal across samples. Purple arrows indicate the mono-nucleosomal DNA and blue arrows indicate the di-nucleosomal DNA (where visible). The pre and post TOP-PCR DNA amounts and % cfDNA (100-700bp) fraction are tabulated.

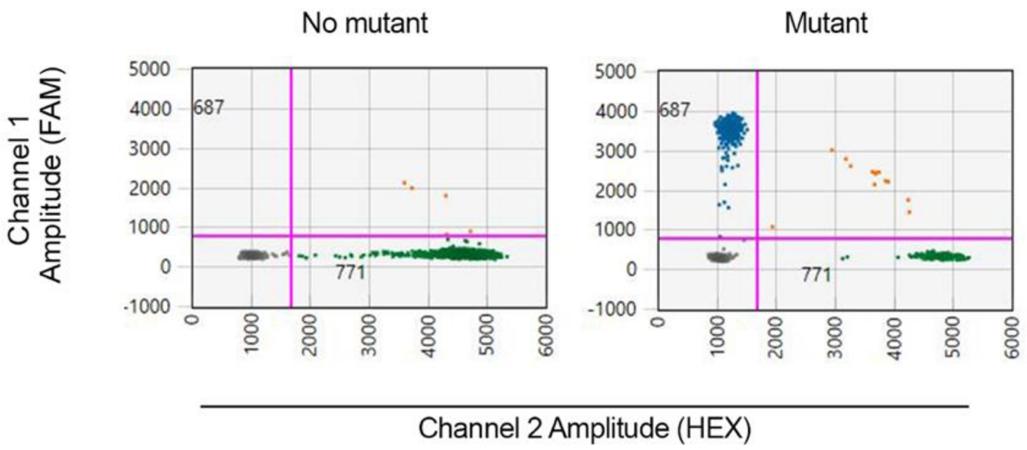

Supplementary Figure S2. **False-positive doublet FAM+/HEX+ droplets in wet-validated ddPCR assay**

Performance of TP53 p. R282W c.844C>T (dHsaMDV2516902; Bio-Rad Laboratories) with wild-type DNA (no mutant) and mutant positive cfDNA. FAM+/HEX- droplets are detected by ddPCR in no mutant control.

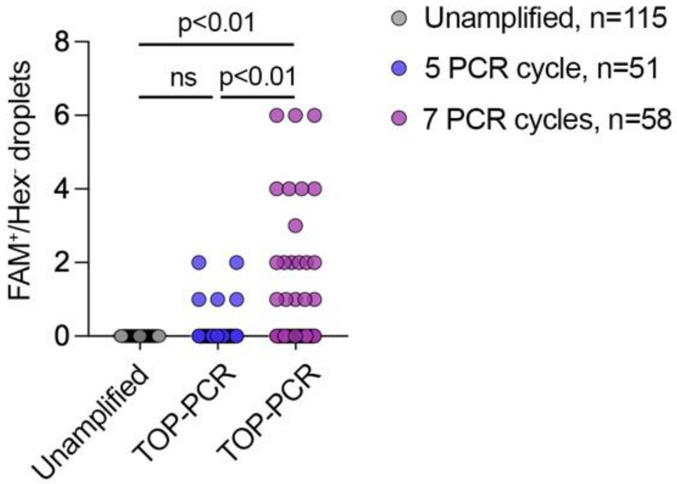

Supplementary Figure S3. TOP-PCR mutation errors increase with cycle number

Number of positive mutant droplets (FAM^+/HEX^-) detected by ddPCR using wild-type (no mutant) cfDNA derived from neonatal human dermal fibroblasts (cfDNA input ranged from 5-60 ng). Data are derived using 20 wet lab validated assays (Supplementary Table 2). Number of PCR runs are indicated for each column. Data are compared using Krukall-Wallis test, with Dunn's multiple comparison test.

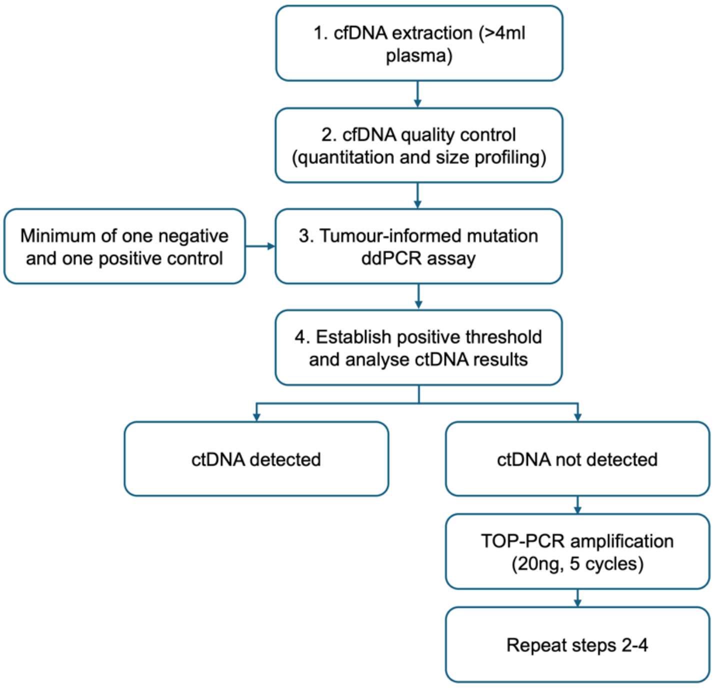

Supplementary Figure S4. Recommended workflow for rare variant detection using TOP-PCR pre-amplification

cfDNA is extracted from at least 4 mL of double-spun plasma using the QIAamp Circulating Nucleic Acid Kit (Qiagen) and eluted in 100μl sterile distilled water. Quality assessment of cfDNA includes DNA quantitation (Qubit 3.0 Fluorometer) and size profiling (TapeStation 4150, Agilent). Tumour-informed ctDNA detection is performed using ddPCR (BioRad QX200 AutoDG, QX600 reader) with 10-40 ng of cfDNA input. A minimum of one negative and one positive control is included in step 3 and in the TOP-PCR reaction. Multiple negative controls are required for each assay to establish a reliable threshold for positivity, against which variant alleles are evaluated.
